# Pharmacological inhibition of poly(ADP-ribose) polymerase-1 modulates resistance of human glioblastoma stem cells to temozolomide

**DOI:** 10.1186/1471-2407-14-151

**Published:** 2014-03-05

**Authors:** Lucio Tentori, Lucia Ricci-Vitiani, Alessia Muzi, Fabio Ciccarone, Federica Pelacchi, Roberta Calabrese, Daniele Runci, Roberto Pallini, Paola Caiafa, Grazia Graziani

**Affiliations:** 1 Department of System Medicine, University of Rome “Tor Vergata”, Via Montpellier 1, 00133 Rome, Italy; 2 Department of Hematology, Oncology and Molecular Medicine, “Istituto Superiore di Sanità”, Viale Regina Elena 299, 00161 Rome, Italy; 3 Department of Cellular Biotechnologies and Hematology, Faculty of Pharmacy and Medicine, “Sapienza” University of Rome, Viale Regina Elena 324, 00161 Rome, Italy; 4 Institute of Neurosurgery, “Università Cattolica del Sacro Cuore”, Largo Agostino Gemelli 8, 00168 Rome, Italy; 5 Pasteur Institute-“Fondazione Cenci Bolognetti”, Piazzale Aldo Moro 5, 00185 Rome, Italy

**Keywords:** Temozolomide, PARP inhibitor, Cancer stem cells, O^6^-methylguanine-DNA-methyltransferase, Chemoresistance

## Abstract

**Background:**

Chemoresistance of glioblastoma multiforme (GBM) has been attributed to the presence within the tumor of cancer stem cells (GSCs). The standard therapy for GBM consists of surgery followed by radiotherapy and the chemotherapeutic agent temozolomide (TMZ). However, TMZ efficacy is limited by O^6^-methylguanine-DNA-methyltransferase (MGMT) and Mismatch Repair (MMR) functions. Strategies to counteract TMZ resistance include its combination with poly(ADP-ribose) polymerase inhibitors (PARPi), which hamper the repair of N-methylpurines. PARPi are also investigated as monotherapy for tumors with deficiency of homologous recombination (HR). We have investigated whether PARPi may restore GSC sensitivity to TMZ or may be effective as monotherapy.

**Methods:**

Ten human GSC lines were assayed for MMR proteins, MGMT and PARP-1 expression/activity, MGMT promoter methylation and sensitivity to TMZ or PARPi, alone and in combination. Since PTEN defects are frequently detected in GBM and may cause HR dysfunction, PTEN expression was also analyzed. The statistical analysis of the differences in drug sensitivity among the cell lines was performed using the ANOVA and Bonferroni’s post-test or the non-parametric Kruskal-Wallis analysis and Dunn’s post-test for multiple comparisons. Synergism between TMZ and PARPi was analyzed by the median-effect method of Chou and Talalay. Correlation analyses were done using the Spearman’s rank test.

**Results:**

All GSCs were MMR-proficient and resistance to TMZ was mainly associated with high MGMT activity or low proliferation rate. MGMT promoter hypermethylation of GSCs correlated both with low MGMT activity/expression (Spearman’s test, P = 0.004 and P = 0.01) and with longer overall survival of GBM patients (P = 0.02). Sensitivity of each GSC line to PARPi as single agent did not correlate with PARP-1 or PTEN expression. Notably, PARPi and TMZ combination exerted synergistic antitumor effects in eight out of ten GSC lines and the TMZ dose reduction achieved significantly correlated with the sensitivity of each cell line to PARPi as single agent (P = 0.01).

**Conclusions:**

The combination of TMZ with PARPi may represent a valuable strategy to reverse GSC chemoresistance.

## Background

Glioblastoma multiforme (GBM) is the most common and aggressive malignant primary brain tumor in adults. Prognosis remains very poor because neoplastic cells invade the brain parenchyma and are naturally resistant to most cytotoxic drugs and radiotherapy [1]. Due to the infiltrative nature of GBM, neurosurgical intervention is not curative. Presently, the current standard of care for patients with newly diagnosed GBM is surgical resection followed by fractionated external beam radiotherapy and systemic temozolomide (TMZ), a methylating agent that crosses the blood–brain barrier [2,3]. However, this treatment modality is not curative and the vast majority of patients experience recurrent disease. Currently, there is no standard treatment for patients with recurrent/resistant GBM, whose median overall survival is only 7 months.

The efficacy of TMZ is limited by the functional status of DNA damage repair systems such as the O^6^-methylguanine-DNA-methyltransferase (MGMT), the Mismatch Repair complex (MMR) and the Base Excision Repair system (BER) [4-6]. In cells with low MGMT levels, unrepaired O^6^-methylguanine mispairs with cytosine or thymine and the resulting mismatches are recognized by the MMR [4]. However, since MMR removes only the base opposite to O^6^-methylguanine, the methylated base persists and mispairs again with thymine. This cycle is repeated with each round of DNA replication, eventually resulting in DNA breaks and cell death. Thus, tumor sensitivity to TMZ requires both low MGMT levels and a functional MMR. About half of GBMs show MGMT promoter hypermethylation with low levels of MGMT expression; in these cases MGMT promoter hypermethylation is associated with prolonged survival of patients treated with TMZ [7]. Deficit in MMR function results in tolerance to TMZ, regardless of MGMT activity levels, and reduced expression of MMR proteins has been frequently reported in human GBMs including those that recur after TMZ [8]. However, TMZ-resistant GBM cells have been described that are MGMT-deficient and MMR-proficient, suggesting that the mechanisms of TMZ resistance are more complex [9].

Among the experimental protocols aimed at increasing TMZ efficacy, an innovative one is based on the association of TMZ with inhibitors of poly(ADP-ribose) polymerase-1 (PARP-1), an enzyme that regulates different cellular processes including DNA repair [5]. Most of the PARP inhibitors (PARPi) in clinical development bind to the catalytic domain of the enzyme and prevent the synthesis of ADP-ribose polymers from NAD^+^ substrate. In preclinical studies, PARPi have been shown to enhance TMZ antitumor activity against GBM human xenografts [10-13] and PARPi are under clinical evaluation in combination with TMZ for the treatment of recurrent or refractory GBM (http://www.clinicaltrials.gov). The mechanism underlying the synergy between PARPi and TMZ relies on the inhibition of the repair of N-methylpurines (i.e., N7-methylguanine and N3-methyladenine) generated by the methylating agent. In fact, these damaged bases normally do no contribute to TMZ cytotoxicity being promptly repaired by the BER system, in which PARP-1 plays a key role. Thus, the enhancing effect exerted by PARPi on temozolomide antitumor activity derives from an increased DNA damage that eventually results in apoptosis and/or growth arrest [5,12].

In addition, PARPi are currently investigated as monotherapy in Breast Cancer gene (BRCA) mutated and homologous recombination (HR) defective tumors, according to a synthetic lethality model. PARP-1 is required for the repair of single strand breaks (SSB); thus, cells with inhibited PARP activity may acquire more unrepaired SSB that, when encounter DNA replication forks, result in fork collapse and DNA double strand breaks (DSB) formation. In normal cells with a functional HR the DSB are repaired, whereas in tumor cells with defective HR the DSB persist and cause cell death [14,15]. Besides mutations or lack of expression of BRCA molecules, deficiency of several other proteins involved in the HR pathway may sensitize cancer cells to PARPi. One example is represented by phosphatase and tensin homolog (PTEN) that is frequently mutated/deleted in GBM [16] and that, among its functions, also regulates transcription of RAD51, an important HR component [17].

Recently, a subset of tumor cells has been identified in GBM that shows stem cell-like features and that is believed to be responsible for tumor initiation and recurrence [18,19]. These cells are generally referred to as GBM stem cells (GSCs). We first demonstrated that GSCs are highly resistant to conventional chemotherapy due to their enhanced DNA repair pathways and drug efflux mechanisms [20]. Therefore, GSCs represent a unique model to investigate whether PARPi may restore sensitivity to TMZ or may be effective as monotherapy in PTEN-deficient GBM. In the present study, we demonstrate that PARP-1 can be efficiently targeted in cancer stem cells in order to increase GBM sensitivity to TMZ and that the potentiating effect induced by PARPi directly correlated with the sensitivity of each cell line to the PARPi used as monotherapy.

## Methods

### Cell cultures

GSCs were isolated from surgical samples of adult patients who had undergone craniotomy at the Institute of Neurosurgery, “Università Cattolica del Sacro Cuore”, School of Medicine, Rome, Italy. Before surgery all patients provided written informed consent according to the Declaration of Helsinki and the research proposal was approved by the Ethical Committee of the “Università Cattolica del Sacro Cuore” (Rome, Italy). The diagnosis of GBM was established on histological examination according to the WHO classification of tumors of the nervous system. Tumor samples were subjected to mechanical dissociation. The resulting cell suspension was cultured in a serum-free medium supplemented with 20 ng/ml EGF and 10 ng/ml FGF-2. Generation of GSCs was defined by the following criteria: *in vitro* formation of primary neurospheres expressing stem cell markers such as CD133, SOX2, Musashi-1 and nestin, capacity of self-renew, ability to co-express astrocytic as well as neuronal phenotypic markers after serum-induced differentiation *in vitro*[20,21]. For immunofluorescence analysis cells were fixed with 4% paraformaldehyde and stained with antibodies directed against SOX2 (goat polyclonal; R&D Systems; 1:200) or Musashi-1 (MAB 2628; R&D Systems; 1:200) or nestin (rabbit polyclonal; Sigma N5413; 1:200). As secondary antibodies, goat anti-rabbit fluorescein isothiocyanate-conjugated IgG (Chemicon; 1:100) were used. Nuclei were counterstained with 4,6-diamidino-2-phenylindole (DAPI) (Vectashield mounting medium with DAPI; Vector Laboratories). Analysis of CD133 was performed by flow-cytometry using an anti-CD133 phycoerythrine conjugated antibody (clone AC133-PE, mouse IgG1, Miltenyi Biotec). All the GSC lines tested in this study were positive for SOX2, Musashi-1 and nestin, whereas they expressed different levels of CD133 (data not shown).

The human GBM cell lines U87 and SJGBM-2 were cultured in DMEM supplemented with 10% fetal calf serum, 2 mM L-glutamine, 100 units/ml penicillin and 100 μg/ml streptomycin (Sigma-Aldrich) at 37°C in a 5% CO_2_ humidified atmosphere. U87 was purchased from ATCC-LGC and SJGBM-2 cell line was a gift from Dr. Peter J. Houghton (St. Jude Children’s Research Hospital, Memphis, TN).

### Drugs

The stock solution of TMZ (100 mM, Sigma-Aldrich) was prepared by dissolving the drug in dimethyl sulfoxide (DMSO). The final concentration of DMSO was always less than 0.5% (v/v) and did not contribute to toxicity. The PARPi GPI 15427 [10-(4-methyl-piperazin-1-ylmethyl)-2H-7-oxa-1,2-diaza-benzo[de]anthracen-3-one, Eisai] stock solution (1 mM) was prepared by dissolving GPI 15427 in 70 mM PBS without potassium [10].

### Drug treatment and analysis of cell growth

Cytotoxicity assays were performed in 96-well plates. GSCs were mechanically dissociated and plated at a density of 2.4×10^4^/ml, in triplicate for each treatment. Compounds were added 3 hours after seeding. Cell viability was estimated after 7 days by the chemiluminescence assay CellTiter-Glo™ (Promega Inc.) following manufacturer’s instructions. Vehicle control (DMSO) luminescence values were averaged and arbitrarily set to 100%. The absolute values of luminescence for each treatment were then normalized with respect to vehicle control and expressed as a percentage.

To evaluate doubling times, mechanically dissociated GSCs were plated in 96-well plates in triplicate and then incubated at 37°C in a 5% CO_2_ incubator. Cell proliferation was monitored by counting cell number at different time points and confirmed by using the CellTiter-Blue Viability Assay (Promega Inc.).

### Western blot analysis

For immunoblot analysis the following primary antibodies were used: monoclonal anti-human MLH1 (clone G168-15, BD Biosciences; 1:500); monoclonal anti-human MSH2 (clone GB12, Calbiochem; 1:1000); monoclonal anti-human MSH6 (clone 44/MSH6, BD Biosciences; 1:500); monoclonal anti-calf PARP-1 (clone C2-10; Trevigen; 1:2000 dilution); monoclonal anti-human PTEN (clone 6H2.1; Cascade Bioscience; 1:1000); goat polyclonal anti-human MGMT (C20; Santa Cruz Biotechnology Inc; 1:1000); rabbit polyclonal anti-human β-tubulin (H-235; Santa Cruz Biotechnology; 1:400). Goat anti-rabbit (Biorad) and goat anti-mouse IgG (Biorad) horseradish peroxidase (HRP)-conjugated secondary antibodies were used at the appropriate dilutions. Immunoreactive bands were detected by enhanced chemoluminescence (ECL) technique using the ECL Plus Western Blotting Substrate (Pierce). Signals were quantified using a Kodak densitometer.

### PARP activity assay

Cells (5×10^6^) were lysed in 0.5 ml of a buffer containing 0.1% Triton X-100, 50 mM Tris–HCl pH 8, 0.6 mM EDTA, 14 mM β-mercaptoethanol, 10 mM MgCl_2_ and protease inhibitors. Proteins (25 μg) were incubated with 2 μCi ^32^P-NAD^+^ (PerkinElmer), 100 μM NAD^+^, 50 mM Tris–HCl, 10 mM MgCl_2_, 14 mM β-mercaptoethanol, in the presence of 10 μg nuclease-treated salmon testes DNA (maximally stimulated activity). After 15 minutes at 30°C the reaction was stopped adding ice-cold trichloroacetic acid 20% (v/v). The radioactivity associated with the acid-insoluble material, corresponding to poly(ADP-ribosyl)ated proteins, was counted on a liquid scintillation counter. PARP activity was evaluated as fmol of ^32^P-NAD^+^/μg of protein.

### MGMT activity assay and bisulfite sequencing analysis of MGMT promoter methylation

Cells (1×10^6^) were lysed in 0.5 ml of a buffer containing 0.5% 3-[(3-cholamidopropyl) dimethylammonio]propanesulfonate, 50 mM Tris–HCl pH 8, 1 mM EDTA, 3 mM dithiothreitol, 100 mM NaCl, 10% glycerol, protease inhibitors and incubated at 4°C for 30 minutes. Various amounts of cell extracts were incubated with 10 μg of calf thymus DNA previously labeled with N-[^3^H]-methyl-N-nitrosourea (18 Ci/mmol; GE Healthcare). MGMT activity was determined by measuring the transfer of [^3^H]-methyl groups from methylated DNA to MGMT and expressed as fmol of methyl groups per mg of proteins in cell extract.

DNA was extracted using DNeasy Blood & Tissue Kit (Qiagen) and converted for bisulfite sequencing analysis using EZ DNA Methylation Kit (Zymo Research) following manufacturer instructions. Bisulfite modified DNA was amplified using the following primer pair: MGMT-C-Bis forward, 5′-GGATATGTTGGGATAGTT-3′; and MGMT-C-Bis reverse, 5′-AAACTAAACAACACCTAAA-3′. Amplification reaction was performed using 5 PRIME HotMasterMix with the following conditions: 95°C for 4 minutes, followed by 42 cycles of 95°C for 30 seconds, 47°C for 30 seconds and 65°C for 30 seconds, with a final extension of 65°C for 5 minutes. Amplified fragments were cloned into the TOPO TA-cloning vector (Invitrogen) and fifteen clones for each GSC line were sequenced by Eurofins MWG Operon service.

### Statistical analysis

The statistical analysis of the differences in drug sensitivity among the cell lines was performed using ANOVA followed by Bonferroni’s post-test and the non-parametric Kruskal-Wallis analysis followed by Dunn’s post-test for multiple comparisons; a P value of less than 0.05 was considered significant. To evaluate whether the combination TMZ + PARPi was synergic, cells were exposed to TMZ alone or in combination with a fixed concentration of GPI 15427. The experiments were performed in quadruplicates and repeated three times. The dose-effect curves were analyzed by the median-effect method of Chou and Talalay using the Calcusyn Software as a non-constant ratio combination (Biosoft). The combination index (CI) indicates a quantitative measure of the degree of drug interaction in terms of synergistic (CI < 1), additive (CI = 1) or antagonistic effect (CI > 1) [22]. Correlation analyses were performed using the Spearman’s rank test and significance was determined according to P values (SSPS software).

## Results

### Analysis of determinants of resistance to TMZ in GSCs

**Figure 1:**
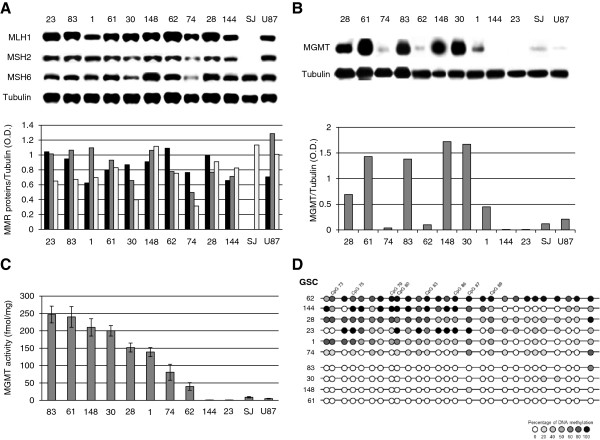
**Analysis of MMR components and MGMT in GSC and GBM cell lines.** Immunoblot analysis of MLH1 (black column), MSH2 (grey column), MSH6 (white column) **(Panel A)** and MGMT proteins **(Panel B)**. Bar graphs represent the mean ratios between the optical densities (O.D.) of the protein of interest and those of tubulin. The results are representative of one out of two experiments with similar results. SJ: SJGBM-2. Panel **C**. Analysis of MGMT activity. MGMT activity is expressed as fmol of methyl groups per mg of total protein and data are the mean (± SD) of three independent experiments. Panel **D**. Analysis of MGMT promoter methylation. Summary of bisulfite sequencing in 10 GSC lines. A total of 27 CpG dinucleotides (CpGs) within the promoter region of MGMT were analyzed and are represented as circles. Each row refers to one individual cell line and circle color indicates the percentage of methylation of each CpG calculated on 15 clones analyzed for each cell line. Closed circles represent fully methylated cytosines, open circles represent fully unmethylated cytosines and grey scale circles represent the indicated percentages of DNA methylation. CpGs 73, 75, 79, 80 (i.e., CpG +95, +113, +135, +137, beginning the numbering at the transcription start site and according to Everhard *et al.*[25]) and CpGs 83, 86, 87, 89 correspond to those that best correlate with gene expression [27].

Ten patient-derived GSC lines (Table 1) and two GBM cell lines (U87 and SJGBM-2) were characterized for the expression of the MMR components MLH1, MSH2 and MSH6, involved in the processing and toxicity of O^6^-methylguanine, and of MGMT, responsible for the removal of the O^6^-methyl adduct. In fact, the lack of expression of one of MMR components and/or the presence of high MGMT levels are associated with resistance to TMZ. The results of Western blot analysis indicated that only the SJGBM-2 cell line was MMR-deficient, lacking MSH2 and MLH1 expression (Figure 1A). Concerning MGMT, the #61, #83, #148 and #30 GSC lines showed the highest expression, whereas #74, #62, #144, #23, U87 and SJGBM-2 lines showed low or barely detectable MGMT protein. The #28 and #1 GSC lines, instead, were characterized by intermediate levels of the repair enzyme (Figure 1B). Analysis of MGMT activity by measuring the ability of cellular extracts to remove methyl adducts from the O^6^ position of guanine in a methylated DNA substrate (Figure 1C) revealed a direct correlation between MGMT activity and protein expression in GCS and GBM cell lines (Spearman’s correlation = 0.87, P < 0.0001, n = 12). In addition, DNA methylation analysis of MGMT CpG island was performed focusing on the region downstream of the transcription start site, which is most commonly investigated by the methylation-specific PCR assay [7,23]. The bisulfite sequencing method was chosen to obtain an unambiguous single-base resolution of DNA methylation status. Notably, MGMT promoter methylation inversely correlated with MGMT activity and expression in GSC lines (Spearman’s correlation = −0.82, P = 0.004 and −0.76, P = 0.01, respectively, n = 10). In particular, the GSC lines with ≥200 fmol/mg MGMT activity were characterized by an unmethylated MGMT promoter (Figure 1D and Table 2). The #74 GSC line showed a modest level of promoter methylation that did not match with the low MGMT expression/activity (Table 2). Considering that the methylation status of regions upstream of the transcription start site may also influence MGMT expression [24,25], DNA methylation analysis of the #74 GSC line was extended to the entire CpG island. However, also upstream regions showed very low levels of DNA methylation (data not shown). The MGMT promoter of U87 GBM cell line was heavily methylated [26].

**Table 1:** Characteristics of the original GBMs from which GSCs were derived

**GCS line**	**WHO classification**	**Tumor location**	**Primary (P) recurrent (R)**	**Overall survival (months)**
**#144**	Grade IV	Temporal	R	19
**#62**	Grade IV	Frontal	R	14
**#1**	Grade IV	Temporal	P	12
**#28**	Grade IV	Frontal	P	11
**#74**	Grade IV	Frontal	P	8
**#83**	Grade IV	Temporal	P	8
**#30**	Grade IV	Frontal	P	7
**#61**	Grade IV	Occipital	P	6
**#23**	Grade IV	Parietal	P	3
**#148**	Grade IV	Parietal	R	1

**Table 2:** Relationship between MGMT expression/activity and MGMT promoter methylation in GSC lines

**GSC**	**MGMT/Tubulin**	**MGMT activity (fmol/mg)**	**CpG 73**	**CpG 75**	**CpG 79**	**CpG 80**	**CpG 83**	**CpG 86**	**CpG 87**	**CpG 89**	**CpG median**^**a**^
**#62**	**0.1**	**40**	83.3	83.3	100	100	100	100	100	100	**100**
**#144**	**0.01**	**1**	50	100	100	100	100	100	83.3	83.3	**83.3**
**#28**	**0.69**	**152**	70	70	70	60	70	50	80	60	**60**
**#23**	**0.01**	**1**	0	100	16.7	100	66.7	100	100	50	**33.3**
**#1**	**0.45**	**139**	70	70	50	60	40	30	60	50	**30**
**#74**	**0.04**	**81**	12.5	25	0	25	0	12.5	62.5	37.5	**12.5**
**#83**	**1.38**	**247**	0	0	0	0	0	0	0	0	**0**
**#30**	**1.67**	**200**	0	0	0	0	0	0	0	0	**0**
**#148**	**1.72**	**210**	0	0	0	0	0	0	14.3	0	**0**
**#61**	**1.43**	**240**	0	0	0	0	0	0	0	0	**0**

^*a*^Median value of DNA methylation of all 27 CpGs analyzed by bisulfite sequencing.

**Figure 2:**
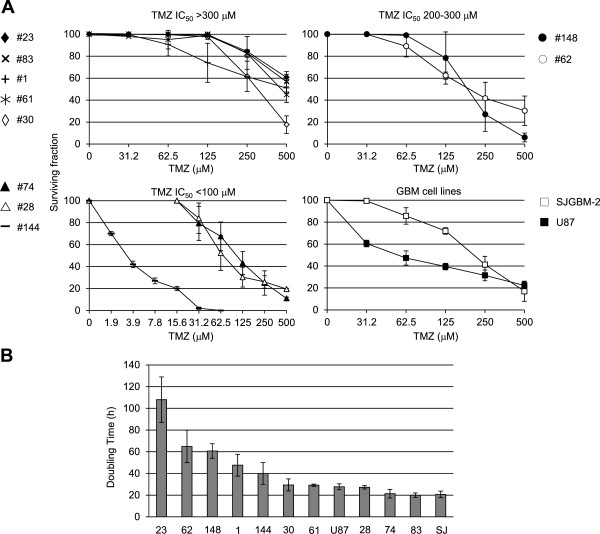
**Sensitivity of GSC lines to TMZ as single agent.** Panel **A**. Chemosensitivity of GSC and GBM cell lines. Tumor growth was evaluated 7 days after drug exposure. Data were plotted in 4 different graphs which gather GSC lines with TMZ IC_50_s >300 μM (#23, #83, #1, #61, #30), with TMZ IC_50_s comprised between 200 and 300 μM (#148, #62), with TMZ IC_50_ < 100 μM (#74, #28, #144) and the two GBM cell lines (SJGBM-2, U87). The results are expressed as survival fraction and are the mean (± SD) of three independent experiments. Panel **B**. Doubling times of GSC and GBM cell lines. Data are the mean (±SD) of three independent determinations.

Chemosensitivity to TMZ was evaluated by measuring ATP production, as a marker of metabolically active cells. Results indicated that most GSC lines with high MGMT activity (≥200 fmol/mg) showed low sensitivity to TMZ, with IC_50_s >300 μM, a value which is well above the peak plasma concentration reached in cancer patients (20–96 μM at a TMZ dose of 200 mg/m^2^) [28]. The #74, #28 and #144 GSC lines were extremely susceptible to TMZ with IC_50_s comprised between 3 and 90 μM, whereas the #62 and #148 GSC lines possessed intermediate sensitivity (200–300 μM) to the methylating agent (Figure 2A). Finally, the GBM U87 cell line, which is MMR-proficient and MGMT-deficient, showed higher sensitivity in comparison with the MMR-deficient SJGBM-2 cell line (Figure 2A). Since actively proliferating cells are more susceptible to TMZ [5,29], the doubling times of the different GSC and GBM lines were measured (Figure 2B) and the results indicated that they ranged from 20 to 108 hours, being #23 the GSC line with the lowest proliferation rate. Interestingly, when GSC lines with similar MGMT activity (<150 fmol/mg) were compared, the cell line with lower proliferative potential resulted more resistant to TMZ (e.g., #23 versus #144; #1 versus #28; #62 versus #74) (Figure 2).

**Figure 3:**
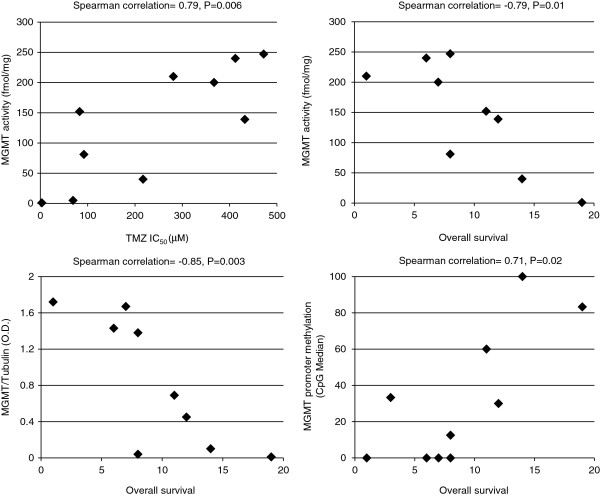
**Correlation analysis of MGMT status and TMZ *in vitro *chemosensitivity of GSCs or patients’ overall survival.** Relationship between MGMT activity and TMZ IC_50_ (*top left*) in GCS and GBM cell lines, except #23 and SJGBM-2; MGMT activity and overall survival (*top right*) or MGMT protein expression and overall survival (*bottom left*) in GCSs except #23; MGMT promoter methylation and overall survival (*bottom right*) in all GSC lines. The Spearman’s correlation coefficients and their significance levels are indicated.

Overall, the response of GSC and GBM cell line to TMZ did not significantly correlate with MGMT activity. However, excluding from the analysis the GSC and GBM cell lines resistant to TMZ for mechanisms unrelated to MGMT status, such as the extremely low proliferation rate (i.e., #23 GSC) or MMR deficiency (i.e., SJGBM-2), sensitivity to TMZ inversely correlated with MGMT activity levels (Spearman’s correlation = 0.79, P = 0.006; n = 10). Excluding from the analysis the slow proliferating #23 GSC line, which is resistant to TMZ even though it lacks MGMT activity, MGMT activity/expression by GSCs inversely correlated with the overall survival of patients from whom the tumor cells were derived (for MGMT activity, Spearman’s correlation = −0.79, P = 0.01; for MGMT protein expression, Spearman’s correlation = −0.85, P = 0.003; n = 9). Noteworthy, promoter methylation status of all the GSC lines directly correlated with patients’ overall survival (Spearman’s correlation = 0.71, P = 0.02, n = 10) (Figure 3).

### Analysis of PTEN and PARP-1 activity/expression and sensitivity to PARPi monotherapy in GSCs

**Figure 4:**
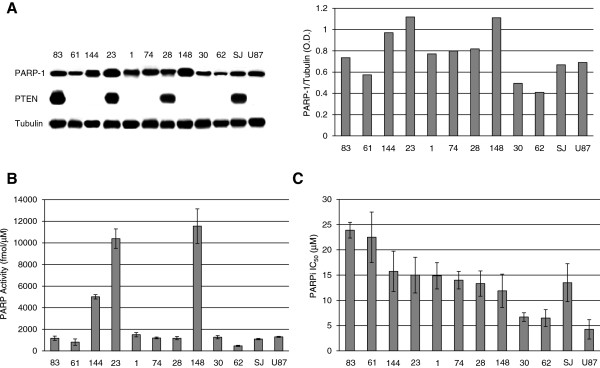
**PARP-1 and PTEN expression in GSC lines and sensitivity to PARPi monotherapy.** Panel **A**. Immunoblot analysis of PARP-1 and PTEN proteins. Bars represent the mean ratios between the O.D. of PARP-1 and those of tubulin. The results are representative of one out of two experiments with similar results. Panel **B**. Analysis of total cellular PARP activity. Maximally stimulated PARP activity was measured in cell extracts obtained from GSC and GBM cell lines in the presence of nuclease-treated salmon testes DNA and ^32^P-NAD^+^ as described in Methods. PARP activity was expressed as fmol ^32^P-NAD^+^/μg of protein and the results are the mean (± SD) of triplicate determinations. Panel **C**. Sensitivity to PARPi. Tumor cells were treated with graded concentrations of GPI 15427 (0.5-50 μM) as single agent and analyzed 7 days after drug exposure. Bars represent the PARPi IC_50_ values and are the mean (± SD) from three independent experiments.

Cell lines were analyzed for PARP-1 and PTEN expression by Western blotting and for sensitivity to PARPi monotherapy. In regard to PARP-1, the #148, #23 and #144 GSC lines showed the highest, whereas #61, #62 and 30# the lowest protein expression (Figure 4A). PARP activity (Figure 4B) in GSC and GBM lines significantly correlated with the expression of PARP-1 protein (Spearman’s correlation = 0.72, P = 0.008, n = 12) that accounts for more than 90% of total cellular poly(ADP-ribosyl)ating activity [12]. On the other hand, PTEN was expressed in #83, #23 and #28 GSC lines, only (Figure 4A); this finding is consistent with the high frequency of PTEN mutations or loss at 10q23 locus reported for GBM [16]. The #83 and #61 GSC were the most resistant ones, whereas #30 and #62 GSC were the most sensitive to the PARPi [P < 0.01, according to ANOVA (α = 0.05) followed by Bonferroni’s post-test and to the non-parametric Kruskal-Wallis analysis followed by Dunn’s post-test] (Figure 4C). Sensitivity of GSC to PARPi did not correlate either with PARP-1 or PTEN expression. The U87 and SJGBM-2 cell lines were characterized by low PARP-1 levels, but they differed in PTEN expression and PARPi sensitivity. In fact, SJGBM-2 cells were PTEN-proficient and more resistant to PARPi than U87 cells (Figure 4).

### PARPi potentiates GSC sensitivity to TMZ

**Figure 5:**
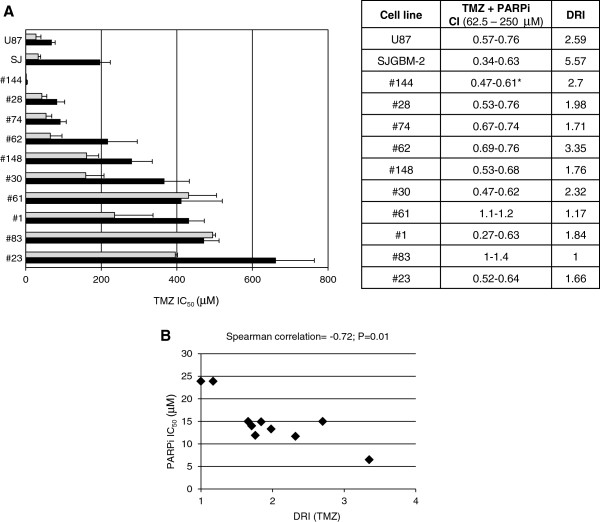
**Modulation of GSC sensitivity to TMZ by PARPi.** Panel **A**. GCS and GBM cell lines were exposed to graded concentration of TMZ as single agent or in combination with a fixed dose of PARPi GPI 15427. Bars represent the TMZ IC_50_ values as single agent (black column) or in combination with PARPi (grey column) and are the mean (± SD) from three independent experiments. The table indicates the values of combination index (CI) for a TMZ concentration range of 62.5-250 (*1.9-7.8 μM in the case of #144 GSC line), evaluated according to the Chou-Talalay method to determine synergy. The DRI values refer to the fold decrease of TMZ IC_50_ obtainable when the methylating agent was combined with the PARPi. Panel **B**. Relationship between PARPi IC_50_ in GSC lines and the DRI (TMZ) in combination with a fixed dose of PARPi. The Spearman’s correlation coefficients and their significance levels are indicated.

With the aim of investigating whether the interruption of N-methylpurine repair by PARPi might revert GSC resistance to TMZ, GSC lines were treated with graded concentrations of TMZ in combination with a fixed dose of the PARPi GPI 15427 that inhibits in living cells more than 80% of PARP activity (5 μM in the case of the #83 and #61 GSC lines, which are the most resistant to PARPi, and 2.5 μM for all the other cell lines) [30]. The drug combination resulted in synergistic effects in 8 out of 10 GSC lines with CI comprised between 0.27 and 0.76 (Figure 5A). Analysis of the dose reduction index (DRI) indicated that addition of PARPi to the methylating agent in GSC lines allowed up to 3.3-fold reduction of TMZ IC_50_. The PARPi increased TMZ efficacy also in both GBM U87 and SJGBM-2 cell lines; the potentiating effect was more pronounced in the latter cells which were more resistant to TMZ as compared to U87 cells because of MMR deficiency (Figure 1A). Interestingly, the DRI of TMZ significantly correlated with the sensitivity of each cell line to the treatment with PARPi as single agent (Figure 5B). In GSCs with the lowest response to PARPi monotherapy, i.e., #83 and #61, PARPi did not enhance the antitumor activity of TMZ.

## Discussion

Although PARPi have been shown to increase the antitumor efficacy of TMZ against a variety of tumor types, the role of these agents as chemosensitizer in GSCs has never been investigated. In the present study, we demonstrate for the first time that PARP-1 can be efficiently targeted in human GSCs in order to increase the sensitivity of these cells to TMZ. More specifically, in eight out of ten GSC lines PARPi allowed up to a 3-fold reduction of TMZ IC_50_s.

We found that all GSCs are MMR-proficient and the resistance to TMZ is mainly caused by an efficient repair of methyl adducts from O^6^-guanine. Seven GSC lines showed TMZ IC_50_s between 200 and 600 μM that are markedly above the peak plasma concentration reached in cancer patients. Only one GSC line (i.e., #23) was resistant to the methylating agent despite MMR-proficiency and lack of MGMT activity/expression. This might be attributed to the extremely low proliferation rate of the #23 line. Cell lines with comparable medium/low MGMT activity but different proliferation rate showed dissimilar TMZ susceptibility. In fact, cytotoxicity related to the processing of O^6^-methylguanine takes place only during the second cycle of DNA replication that follows adduct generation. The presence of a non-proliferating compartment in the tumor mass may limit the efficacy of TMZ as monotherapy even in the case of malignancies with functional MMR and low MGMT activity. On the other hand the killing effect, deriving from interruption of BER-mediated repair process of N-methylpurines by PARPi, may occur even during the first round of cell division and in the absence of DNA synthesis [29]. Indeed, PARPi potentiated the sensitivity to TMZ also in slow proliferating GSCs. Excluding the slow proliferating #23 GSC line, the anti-tumor effects of TMZ in MMR-proficient cell lines inversely correlated with MGMT activity/expression at a statistically significant level.

Epigenetic silencing of MGMT expression is regarded as a prognostic factor and valuable predictive marker of TMZ efficacy in GBM [7,31]. CpG methylation in the MGMT promoter of the GSC lines ranged from 0% to 100% and all GSC lines with a MGMT promoter containing 0% methylated CpGs showed very high enzymatic activity (≥200 fmol/mg) and resistance to TMZ. Interestingly, the percentages of CpG methylation in the promoter of GSCs significantly correlated with patients’ overall survival. Although statistical analysis indicated an inverse correlation between MGMT promoter methylation and MGMT activity, in #74 GSC the entire CpG island was mainly demethylated despite low MGMT activity (<100 fmol/mg)/protein expression and high TMZ sensitivity (IC_50_ < 100 μM). This evidence may depend on a DNA methylation-independent mechanism based on altered chromatin configuration and gene silencing described for MGMT [32]. In this case the sole analysis of MGMT promoter methylation status would have led to underestimation of tumor chemosensitivity. Our findings are in agreement with a recent report indicating that determination of both promoter methylation and protein expression is required for an optimal assessment of MGMT status [33]. Nevertheless, the short overall survival of the patient, from whom #74 GSC derives, appears to match with the promoter demethylated pattern, thus confirming the importance of the MGMT promoter methylation as prognostic factor [34]. Interestingly, excluding the GSC line resistant to TMZ as a consequence of the extremely low proliferation rate (i.e., #23), also MGMT activity/expression was inversely related with patients’ overall survival at a statistically significant level.

Sensitivity of tumors to PARPi monotherapy has been recently linked to PARP-1 expression, suggesting that for a NAD^+^ competitor to be functional, enough PARP-1 target must be available to bind DNA strand breaks and synthesize poly(ADP-ribose) [35]. The susceptibility of GSC lines to PARPi did not correlate with PARP-1 protein levels or with total cellular PARP activity. Actually, the most resistant lines (i.e. #83 and #61) were characterized by PARP activity comparable to that of the most sensitive GSC lines (i.e. #30 and #62). These data suggest that PARP-1 expression itself is not a limiting factor for PARPi efficacy in GSC lines.

Alterations in PTEN gene on 10q23 at the level of loss of heterozygosity, mutation and methylation have been identified in at least 60% of GBMs [16]. In agreement with these findings, PTEN was not expressed in the majority (7 out of 10) of the GSC lines tested. Lack of PTEN expression has been recently associated with increased sensitivity to PARPi monotherapy according to a synthetic lethality model [36]. Even though the highly sensitive #30 and #62 GSC lines did not express PTEN, no statistically significant correlation was found between PARPi IC_50_ of all the GSC lines and PTEN protein expression. These results are consistent with data reported for prostate cancer in which PTEN status did not behave as a biomarker for HR function and response to PARPi [37]. Since the lack of PTEN protein expression in GSC or GBM cells may derive from PTEN gene mutations or from deletion of chromosome band 10q23 involving other genes, we cannot exclude that the mutation status and copy number changes might have different roles in the sensitivity to PARPi.

Treatment with the PARPi enhanced TMZ efficacy in all but two GSC lines and the potentiating effect directly correlated with sensitivity of each cell line to the PARPi used as single agent. In fact, the #30 and #62 GSC lines, which were sensitive to GPI 15427, became highly vulnerable to the combination of TMZ and PARPi. The two GSC lines (#83 and #61) in which TMZ and PARPi association did not result in synergistic effects were the most resistant to PARPi monotherapy and were poorly responsive to TMZ as well. The PARPi used in our study GPI 15427 and its analogue E7016 have shown ability to cross the blood–brain barrier and chemo-radiosensitizing activity in preclinical models of glioblastoma [10,11]. E7016 is currently under clinical investigation in association with TMZ for solid tumors comprising gliomas (phase I, NCT01127178) and metastatic melanoma including cerebral metastases (phase II, NCT01605162) (http://www.clinicaltrials.gov). The PARPi provoked a remarkable dose reduction of TMZ in the MMR-deficient SJGBM-2 cells, and such reduction was higher than that obtained in the MMR-proficient U87 GBM cells. This finding suggested that the chemosensitizing effect is maximal in tumor cells tolerant to O^6^-methylguanine, which is regarded as the main cytotoxic adduct generated by TMZ. In MMR-deficient GBM cells inhibition of PARP catalytic activity converts N-methylpurines in cytotoxic lesions since they are no longer repaired by BER [38].

It was previously demonstrated that tumor clones selected for resistance to PARPi plus TMZ expressed lower levels of PARP-1 as compared to parental sensitive cells [39]. In our GSC model PARP-1 expression/activity did not appear to influence chemosensitization mediated by the PARPi. Indeed, we have recently found that inhibition of PARP-1 function increased the antitumor activity of platinum compounds or topoisomerase I poisons even in the presence of low PARP-1 expression [40,41].

## Conclusions

In conclusion, the combination of TMZ with PARPi is a valuable strategy to counteract chemoresistance of GSCs which contributes to treatment failure and tumor recurrence in GBM patients.

## Competing interests

The authors declare that they have no competing interests.

## Authors’ contributions

LT and LRV conceived the idea; AM, FC, RC, FP and DR performed the experiments; GG, LT and LRV designed the experiments. GG, LT, RP PC and LRV analyzed and discussed data. GG and LT wrote the manuscript. All authors read and approved the manuscript.

## Pre-publication history

The pre-publication history for this paper can be accessed here:

http://www.biomedcentral.com/1471-2407/14/151/prepub
